# LINC01783 facilitates cell proliferation, migration and invasion in non-small cell lung cancer by targeting miR-432-5p to activate the notch pathway

**DOI:** 10.1186/s12935-021-01912-0

**Published:** 2021-04-26

**Authors:** Yanchao Deng, Liwei Zhang, Ruiying Luo

**Affiliations:** 1 Department of Thoracic Surgery, First Affiliated Hospital of Xinjiang Medical University, Urumqi, 830054 Xinjiang China; 2 General Surgery, The Second Hospital of Lanzhou University, Lanzhou, 730030 Gansu China

**Keywords:** LINC01783, MiR-432-5p, DLL-1, Non-small cell lung cancer

## Abstract

**Background:**

Non-small cell lung cancer (NSCLC) is a common malignancy around the globe. Increasing long non-coding RNAs (lncRNAs) have been confirmed to be associated with the progression of cancers, including NSCLC. Long intergenic non-protein coding RNA 1783 (LINC01783) is a novel lncRNA and its regulatory function as competing endogenous RNA (ceRNA) has not been studied in NSCLC.

**Methods:**

RT-qPCR measured the expression level of LINC01783 in NSCLC cells. CCK-8, EdU, transwell and wound healing assays were conducted to detect cell proliferation, migration and invasion in NSCLC. The relationship between miR-432-5p and LINC01783 along with delta like 1 (DLL-1) was illustrated by RNA pull down, RIP and luciferase reporter assays.

**Results:**

LINC01783 was found remarkably increased in NSCLC cell lines, and down-regulation of LINC01783 suppressed cell proliferation, migration and invasion. Then, we discovered Notch pathway was related to the progression of NSCLC, and DLL-1 expression was reduced by LINC01783 knockdown. Furthermore, DLL-1 overexpression could counteract the suppressive effects of LINC01783 down-regulation on the growth of NSCLC cells. MiR-432-5p was observed to be the mutual miRNA that could bind with both LINC01783 and DLL-1. Overexpression of miR-432-5p inhibited DLL-1 expression. In the rescue assays, miR-432-5p depletion offset the impacts of LINC01783 knockdown, and then DLL-1 silence recovered the influence of miR-432-5p down-regulation on NSCLC cell growth.

**Conclusion:**

LINC01783 aggravates NSCLC cell growth by regulating Notch pathway and sponging miR-432-5p, being a potential target in the treatment for NSCLC.

**Supplementary Information:**

The online version contains supplementary material available at 10.1186/s12935-021-01912-0.

## Background

Lung cancer is the top cause of cancer-related death cases in the USA [1]. The statistics showed that 57% lung cancer patients at metastatic stage were with a dramatically low 5-year survival rate of 5% while the survival rate for localized-stage patients is 57% [1]. NSCLC is the most common form of lung cancer and covers more than 80% of lung cancer-related death [2, 3]. Clinically, only a minority of NSCLC patients are diagnosed at an early stage (Stage I or II), at which point the tumor can be subjected to surgical resection [4]. The majority of lung cancer patients are diagnosed as locally advanced or metastatic disease (Stage III or IV) at which point the surgery may not be an option. At present, radiation therapy and conventional chemotherapy remain the main treatment methods for lung cancer patients [5]. In recent years, the targeted therapies and immunotherapy for NSCLC treatment has been increasingly investigated. Of note, the long non-coding RNAs (lncRNAs) have been involved in the molecular diagnosis, targeted therapy, and predicting prognosis of lung cancer [6]. Although great advancements have been achieved in the diagnosis and treatment, the overall survival rate is still very low and the recurrence possibility is increasing due to metastasis and chemoresistance [7, 8]. Therefore, it is extremely necessary to find out effective biomarkers for NSCLC treatment.

Long non-coding RNAs (lncRNAs), a group of non-coding RNAs with over 200 nucleotides in length, lack protein-coding capacities but can regulate expression of genes at the transcriptional or post-transcriptional level [9]. For example, lncRNA PTAR is up-regulated in human NSCLC cells [10] and serves as a marker of NSCLS for diagnosis.

LncRNAs have been reported to function as a ceRNA to promote cancer progression via competitively sponging miRNAs to mediate mRNAs expression. A large body of evidence demonstrates that ceRNA networks have consequences for various types of cancers including NSCLC. For instance, C5orf66-AS1 promoted cell growth in cervical cancer through sponging miR-637 to regulate RING1 [11]. HOXA-AS2 down-regulation inhibited the chemoresistance of acute myeloid leukemia through sponging miR-520c-3p to elevate S100A4 [12]. LINC01783 is a novel lncRNA which has been rarely studied. In 2020, the promoting role of LINC01783 in cervical cancer via regulating miR-199b-5p/GBP1 has been verified [13]. However, the function of LINC01783 in the progression of NSCLC remains unknown and is worthwhile to be investigated.

Our current study aimed to explore the underlying role and regulatory mechanism of LINC01783 via Notch pathway in NSCLC progression.

## Methods

### Cell lines

Human lung bronchial epithelial cell line BEAS-2B and human NSCLC cell lines (NCI-H460, NCI-H1975, NCI-H1299 and A-549) were all procured from the ATCC (Manassas, VA, USA) for this study. BEAS-2B cell line was cultured in BEGM (Lonza/Clonetics Corporation, Walkersville, MD, USA). A-549 cell line was cultured in F-12K Medium, and the other cell lines were cultured in RPMI-1640 Medium (Gibco, Grand Island, NY, USA). All media were added with 10% fetal bovine serum (FBS; Gibco). Cell culture was undertaken in an incubator with 5% CO_2_ at 37 °C.

### Total RNA extraction and reverse transcription qPCR (RT-qPCR)

TRIzol Reagent (Invitrogen, Carlsbad CA, USA) was commercially acquired to extract total RNA, as per the user guide. Then, PrimeScript Reverse Transcriptase Kit (Takara, Shiga, Japan) was used for cDNA synthesis. Expressions of genes were analyzed by qPCR with SYBR Green PCR Kit (Takara), and calculated based on the comparative change-in-cycle method (2^−ΔΔCt^). GAPDH or U6 was utilized as the internal reference.

### Plasmid transfection

NCI-H460 and A-549 cells were plated in 6-well plates and transfected with specific shRNAs (GenePharma, Shanghai, China) targeting LINC01783 or DLL-1 for 48 h, using Lipofectamine 3000 (Invitrogen). The negative control shRNAs (sh-NCs) were used for comparison. The full-length of LINC01783 or DLL-1 sequences were sub-cloned into the pcDNA3.1 vectors (Invitrogen) for overexpression in further assays. Besides, the miR-432-5p inhibitors, miR-432-5p mimics and NCs were synthesized by Ribobio (Guangzhou, China) for transfection. Three independent repeats were conducted.

### Cell counting kit-8 (CCK-8) assay

5 × 10^3^ transfected NCI-H460 and A-549 cells were collected for CCK-8 assay. Cells were treated in 96-well plates with 10 μL CCK-8 solution (Dojindo, Kumamoto, Japan) for 2 h. The OD value was examined by the spectrophotometer (Thermo Fisher Scientific, Waltham, MA, USA) at 450 nm. Three independent repeats were conducted.

### EdU assay

After transfection, NCI-H460 and A-549 cells (1 × 10^4^) were seeded in 96-well plates and then treated with EdU assay Kit (Ribobio) at 37 °C for 2 h. Next, cell samples were treated with DAPI solution at room temperature for 5 min, followed by observation under a fluorescence microscope (Olympus, Tokyo, Japan). Three independent repeats were conducted.

### Transwell assay

Transwell assays were carried out by using transwell chambers with or without Matrigel to evaluate the invasive or migratory capacity of NSCLC cells. 2 × 10^4^ cells of NCI-H460 and A-549 were seeded in the upper chambers with serum-free medium of transwell insert (Corning Incorporated, Corning, NY, USA). 100% complete medium was added into lower chambers. After being fixed by 4% paraformaldehyde, the cells that invaded or migrated into the lower chambers were visualized by crystal violet staining. Five random fields were chosen and observed by optical microscope (Olympus). Three independent repeats were conducted.

### Wound healing

Transfected NCI-H460 and A-549 cells were incubated in 6-well plates for reaching 100% cell confluence. Cells were then wounded using 200-μL pipette tip in the middle and cultured in serum-free medium under the circumstance of 37 °C and 5% CO_2_. The wound healing was detected at 0 and 24 h for analyzing cell migration. Three independent repeats were conducted.

### In vivo subcutaneous xenograft tumor model

8 nude mice (female, 4-week-old), purchased from the Experimental animal center of Chinese Academy of Sciences, were randomly separated into two groups with 4 in control group and 4 in the experimental group. A-549 cells (0.1 × 10^7^/mL) transfected with sh/NC or sh/LINC01783#1 were subcutaneously injected in the control groups or experimental groups correspondingly. Tumor growth was monitored every four days. After 28 days, tumors were excised and collected for further analysis. This assay has been approved by the Animal Ethics Committee of First Affiliated Hospital of Xinjiang Medical University.

### Luciferase reporter assay

To confirm the pathway involved, several pathways were selected to implement luciferase reporter assays with Cignal Finder Signal Transduction 45-pathway Reporter Array (No-CCA-901L, No-336821). To analyze NOTCH pathway activity, NCI-H460 and A-549 cells were co-transfected with sh/LINC01783#1 or sh/NC, and RBP-Jκ luciferase reporter plasmid using RBP-Jκ reporter kit (SABiosciences, Frederic, MD, USA). In addition, cells were co-transfected with indicated plasmids and the pmirGLO dual-luciferase reporter vectors (Promega, Madison, WI, USA) which contained the wild-type and mutated fragments of DLL-1-3′UTR or LINC01783. After 48 h of co-transfection, Dual-luciferase reporter assay system (Promega) was used for analysis. Three independent repeats were conducted.

### Subcellular fractionation

Subcellular fractionation assay of NCI-H460 and A-549 cells was carried out using PARIS™ Kit (Invitrogen). Cell samples were first rinsed in PBS, and then subjected to cell fractionation buffer. The cellular distribution of LINC01783, U6 and GAPDH were individually monitored by RT-qPCR. Three independent repeats were conducted.

### FISH

NCI-H460 and A-549 cell samples were washed in PBS for being incubated with LINC01783-specific FISH probes (Ribobio) in the hybridization buffer. DAPI solution was subsequently used to stain cell nucleus. Cells were imaged by use of fluorescence microscope (Olympus). Three independent repeats were conducted.

### RNA binding protein immunoprecipitation (RIP)

For RIP assay, Magna RIP™ RNA-Binding Protein Immunoprecipitation Kit (Millipore, Bedford, MA, USA) was commercially acquired and employed as instructed. The human Ago2 antibodies and NC IgG antibodies were purchased from Cell Signaling Technology (Danvers, MA, USA). Cell lysates were cultured in RIP buffer with antibodies bound to magnetic beads for 6 h at 4 °C, then, RNAs in the precipitation comprising of magnetic beads, antibodies, proteins and RNAs were abstracted to be analyzed by RT-qPCR. Three independent repeats were conducted.

### RNA pull down

RNA pull down assay was carried out via Pierce Magnetic RNA–Protein Pull-Down Kit (Thermo Fisher Scientific). The cell proteins were mixed with biotin-labeled (Bio-) miR-432-5p probes containing LINC01783 or DLL-1 binding sites. After the addition of magnetic beads, the mixture was eluted and RNAs were analyzed by RT-qPCR. Three independent repeats were conducted.

### Statistical analyses

Experimental results of each assay were displayed as mean ± SD. The data analysis between groups was achieved by one-way/two way ANOVA or Student’s t-test, using GraphPad PRISM 6 (GraphPad, San Diego, CA, USA). When P was < 0.05, data were collected for analysis.

## Results

### LINC01783 is overexpressed and promotes cell proliferation and migration in NSCLC

**Fig. 1:**
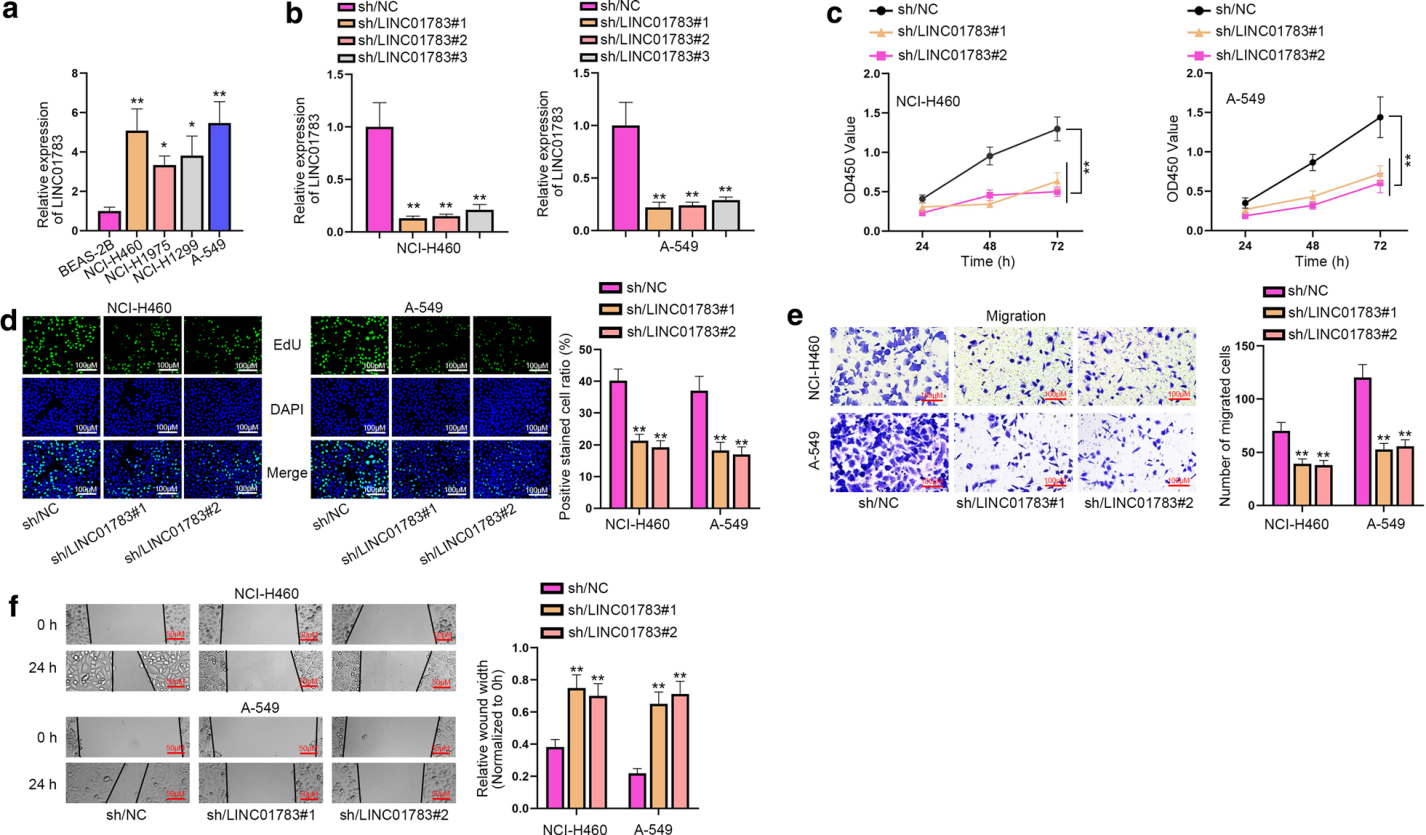
LINC01783 is overexpressed and promotes cell proliferation and migration in NSCLC. **a** LINC01783 expression was detected by RT-qPCR in NSCLC cell lines (NCI-H460, NCI-H1975, NCI-H1299 and A-549) and normal lung epithelial cell line (BEAS-2B) (One-way ANOVA, Tukey). **b** The knockdown efficiency of sh/LINC01783#1/2/3 in NCI-H460 and A-549 cells was appraised by RT-qPCR (One-way ANOVA, Tukey). **c**, **d** CCK-8 and EdU assays assessed proliferation in cells with sh-LINC01783#1/2 (One-way ANOVA, Dunnett). **e**, **f** Cell migration capability was evaluated by transwell and wound healing assays after the knockdown of LINC01783 (One-way ANOVA, Dunnett). *P < 0.05, **P < 0.01

To explore the biological role of LINC01783 in NSCLC cells, RT-qPCR was performed to measure LINC01783 expression in NSCLC cell lines. It was revealed that LINC01783 was conspicuously overexpressed in NSCLC cell lines (NCI-H460, NCI-H1975, NCI-H1299, and A-549) compared with normal lung epithelial cell line (BEAS-2B) (Fig. 1a). Considering that BEAS-2B cell line was infected with an adenovirus 12-SV40 virus hybrid (Ad12SV40) and cloned according to product information on the ATCC, we measured the expression of LINC01783 in BEAS-2B cells to determine whether Ad12-SV40 makes a difference on LINC01783 expression. The results showed that Ad12SV40 has little effect on LINC01783 expression in BEAS-2B cells (Additional file 1: Figure S1A). In addition, NCI-H460 and A-549 cells were used for further investigation because the expression of LINC01783 in NCI-H460 and A-549 cells were higher than that in NCI-H1975 and NCI-H1299 cells. Then, sh/LINC01783#1/2/3 were transfected into NSLCL cells and LINC01783 was significantly silenced in all groups, verifying the knockdown efficiency of sh/LINC01783#1/2/3 plasmids (Fig. 1b). Then, sh/LINC01783#1 and sh/LINC01783#2 were applied for further study. Results of CCK-8 and EdU assays exhibited that proliferative ability of NSCLC cells were terribly decreased by LINC01783 silence since both OD450 value and number of EdU positive cells were reduced overtly after LINC01783 was downregulated in NSCLC cells (Fig. 1c, d). At the same time, cell migration was detected through transwell migration and wound healing assays. The results showed that the number of migrated cells was dramatically decreased in the group of sh-LINC01783-transfected cells while the relative wound width in wound healing assay was apparently increased compared with NC groups (Fig. 1e, f). Consistently, transwell assay showed that number of invaded cells was distinctly reduced in sh/LINC01783#1/2-transfected groups compared with NC groups (Additional file 1: Figure S1B). Taken together, LINC01783 enhances cell proliferation, migration and invasion in NSCLC. To further explore the function of LINC01783 in NSCLC, nude mice model was established. Results showed that tumor growth in nude mice injected with transfected NSCLC cells with sh/LINC01783#1 was evidently inhibited compared with that in the control group in terms of both volume and weight (Additional file 1: Figure S1C). In conclusion, LINC01783 is up-regulated in NSCLC cells and promotes NSCLC progression.

### LINC01783 regulates Notch pathway and facilitates the progression of NSCLC cells via upregulating DLL-1

**Fig. 2:**
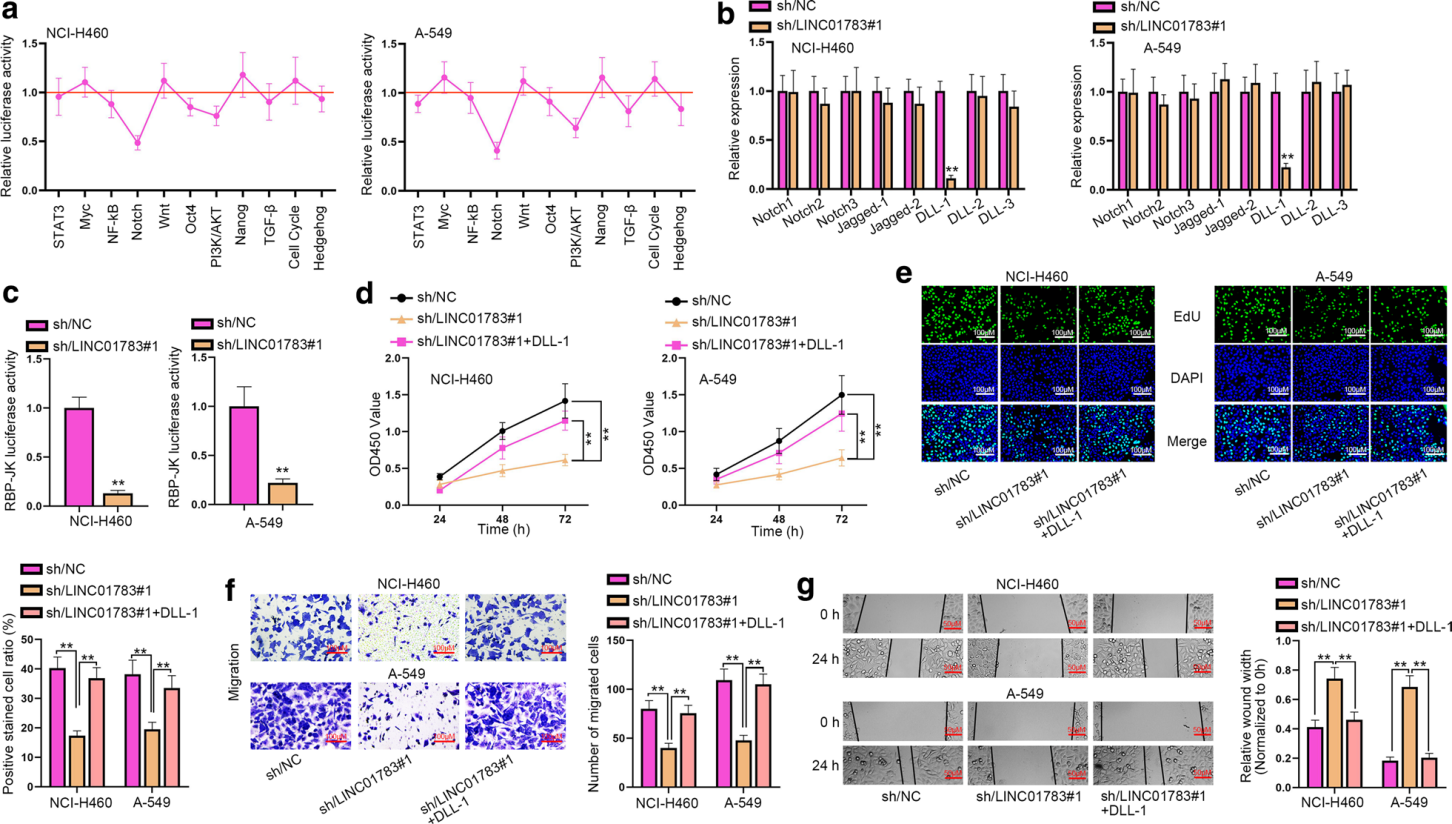
LINC01783 regulates Notch pathway and facilitates the progression of NSCLC cells via upregulating DLL-1. **a** Luciferase reporter assays examined the luciferase activity of each pathway (One-way ANOVA, Tukey). **b** The expressions of receptors and ligands in Notch pathway were measured by RT-qPCR in the cells with sh/LINC01783#1 (Student’s t-test). **c** RBP-Jκ reporter assays examined the luciferase activity of Notch pathway (Student’s t-test). **d**–**g** CCK-8 (**d**), EdU (**e**), transwell (**f**) and wound healing (**g**) assays measured cell proliferation (**d**, **e**) and migration (**f**, **g**) in NCI-H460 and A-549 cells transfected with sh/NC, sh/LINC01783 and DLL-1 vector (One-way ANOVA, Tukey). **P < 0.01

Pathways were often associated with the development of multiple cancers. Hence, we inferred that certain pathways were involved in the development of NSCLC. We detected the luciferase activity of some pathways in NSCLC cells. According to the data of luciferase reporter assays, we found that the activity of Notch pathway was the lowest when LINC01783 expression was inhibited (Fig. 2a). Additionally, we utilized Western Blot assay to analyze the expression of Notch pathway related proteins in NCI-H460 and A-549, finding that expressions of these proteins were significantly diminished when LINC01783 was knocked down (Additional file 1: Figure S1D). Then we detected the expression of receptors and ligands in Notch pathway by using RT-qPCR. The results presented that only DLL-1 expression was remarkably cut down by down-regulated LINC01783 (Fig. 2b). Then, data of RBP-Jκ luciferase reporter assays exhibited that LINC01783 silence dwindled the RBP-Jκ luciferase activity (Fig. 2c). Hence, Notch pathway was determined to be the pathway involved in LINC01783-related mechanism in NSCLC. Then, to corroborate whether LINC01783 functioned through DLL-1, rescue assays were performed. Down-regulation of LINC01783 suppressed proliferation of NCI-H460 and A-549 cells, but then overexpression of DLL-1 recovered the inhibitory effects induced by silenced LINC01783 on proliferative capacities of NSCLC cells according to the results of CCK-8 and EdU assays (Fig. 2d, e). Falling tendency of cell migration and invasion imposed by LINC01783 knockdown was reversed by up-regulation of DLL-1 in transwell and wound healing assays (Fig. 2f, g, Additional file 1: Figure S1E). In summary, LINC01783 activates Notch pathway and boosts the progression of NSCLC cells via modulating DLL-1 expression.

### MiR-432-5p is targeted by LINC01783 in NSCLC cells

**Fig. 3:**
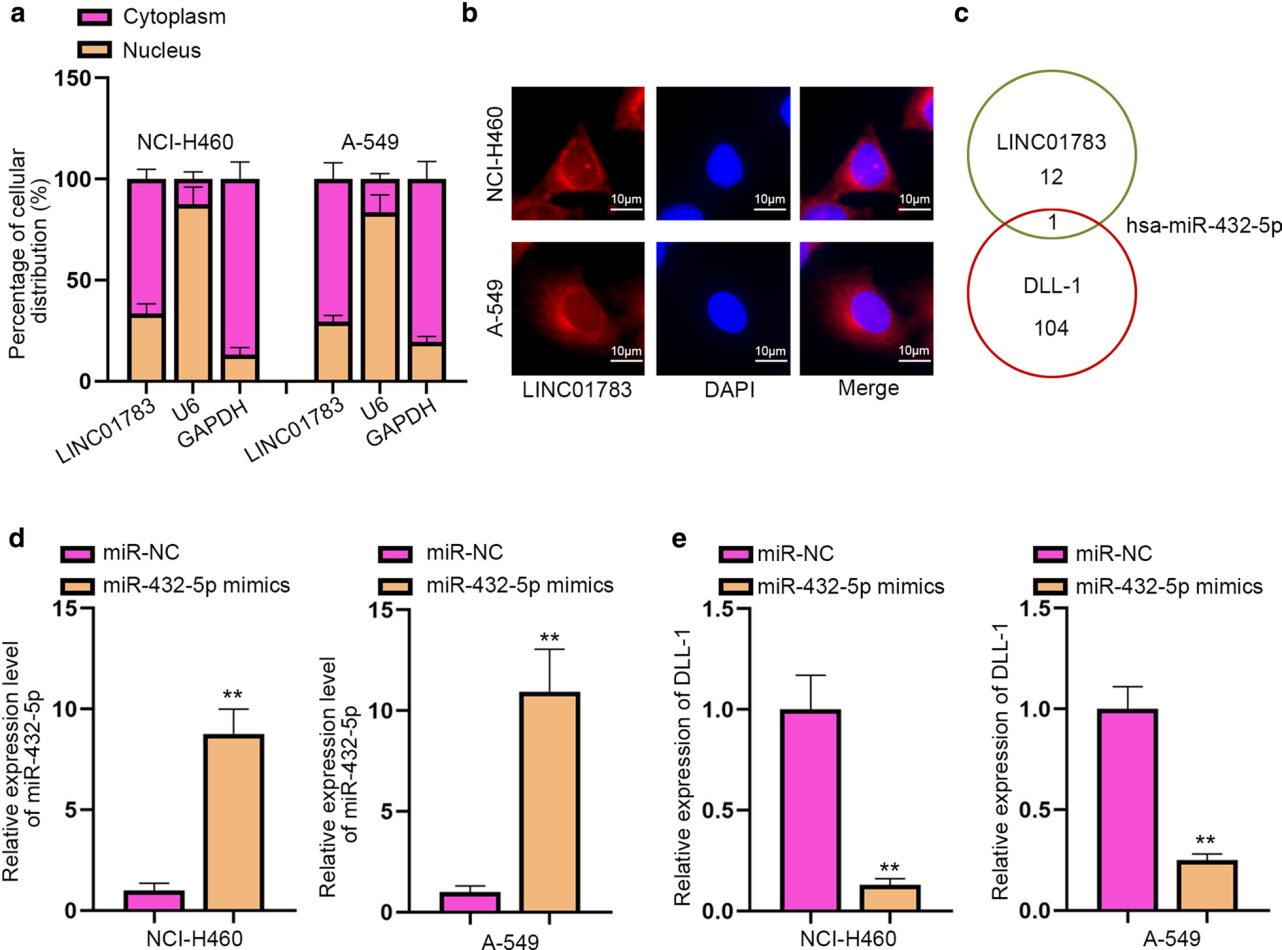
MiR-432-5p is targeted by LINC01783 in NSCLC. **a**, **b** Subcellular fractionation (Student’s t-test) and FISH assays were performed to determine the distribution of LINC01783 in NCI-H460 and A-549 cells. **c** The Venn diagram showed that LINC01783 and DLL-1 shared one mutual miRNA (miR-432-5p) predicted by the starBase database (http://starbase.sysu.edu.cn/index.php). **d** The overexpression efficiency of miR-432-5p mimics in NSCLC cells were evaluated (Student’s t-test). **e** DLL-1 expression was evaluated by RT-qPCR in cells with miR-432-5p mimics (Student’s t-test). **P < 0.01

Then, we performed subcellular fractionation and FISH assays to judge the subcellular distribution of LINC01783 in NSCLC cells. Data revealed that LINC01783 was mainly localized in the cytoplasm (Fig. 3a, b). Therefore, we hypothesized that LINC01783 might function as a ceRNA. Then, after searching starBase (http://starbase.sysu.edu.cn/index.php) database, we found that miR-432-5p was the only shared miRNA of LINC01783 and DLL-1 though LINC01783 could bind with 13 miRNAs and DLL-1 could bind with 105 miRNAs (Fig. 3c). MiR-432-5p mimics were transfected into cells to enhance miR-432-5p expression (Fig. 3d). Subsequently, DLL-1 expression was detected to be cut down by up-regulation of miR-432-5p (Fig. 3e). Altogether, miR-432-5p is targeted by LINC01783 and negatively modulates DLL-1 expression.

### LINC01783 modulates miR-432-5p to regulate DLL-1 expression

**Fig. 4:**
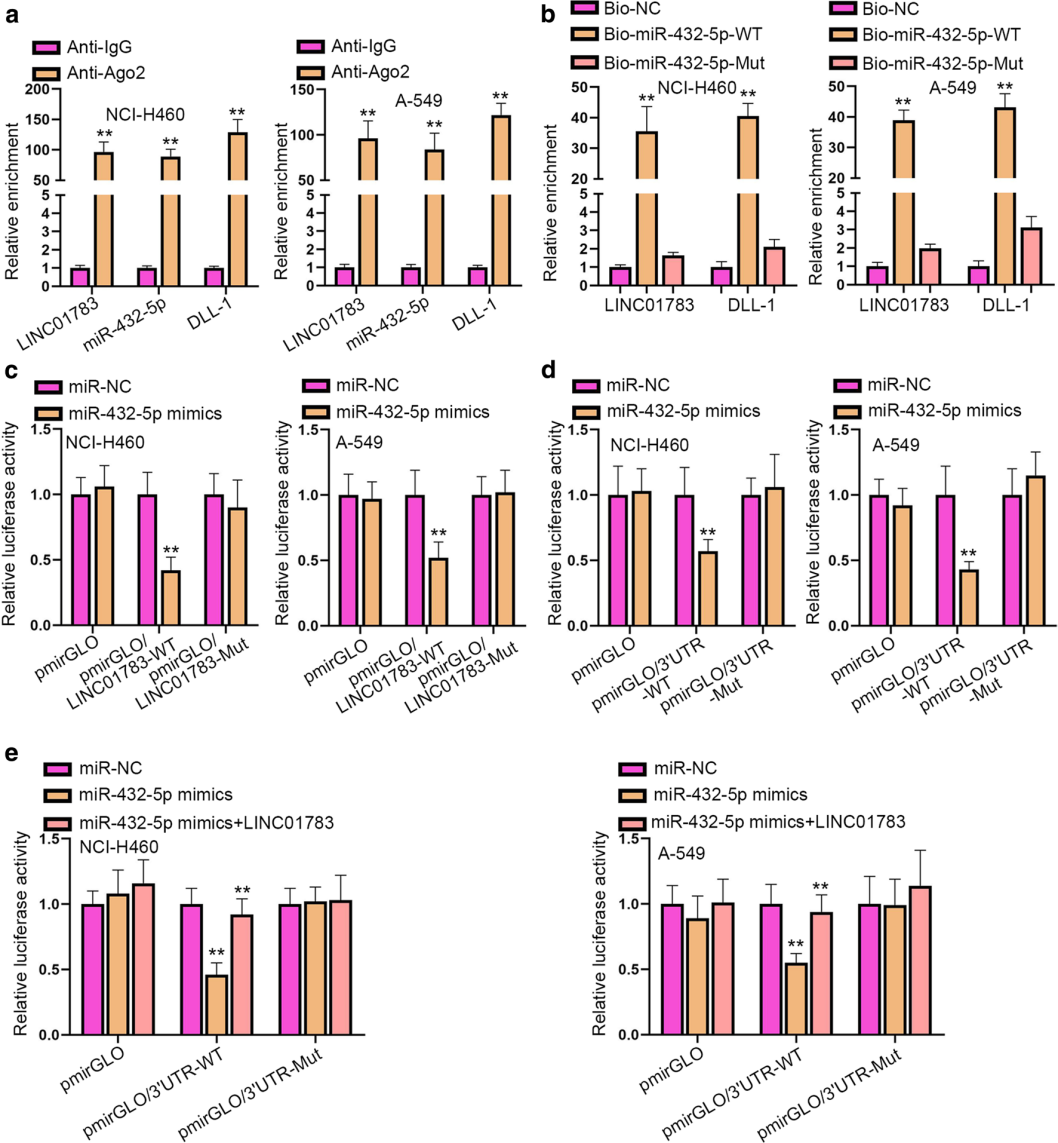
LINC01783 modulates miR-432-5p to regulate DLL-1 expression. **a**, **b** RIP assays (Student’s t-test) and RNA pull down assays (One-way ANOVA, Tukey) were used to validate the relationship between LINC01783, miR-432-5p and DLL-1. **c**, **d** Luciferase reporter assays were carried out to examine the binding sites between miR-432-5p with LINC01783 as well as DLL-1 (Two-way ANOVA, Tukey). **e** The competing relationship between LINC01783 and DLL-1 was evaluated by luciferase reporter assays (Two-way ANOVA, Tukey). **P < 0.01

Next, we used mechanism assays to validate the relationship among LINC01783, miR-432-5p and DLL-1. As shown in Fig. 4a, RIP data disclosed that LINC01783, miR-432-5p and DLL-1 were enriched in Ago2 precipitates rather than in IgG ones. Before RNA pull down and luciferase reporter assays, the binding sites of LINC01783, miR-432-5p and DLL-1 were mutated accordingly (Additional file 1: Figure S1F). RNA pull down assays manifested that Bio-miR-432-5p-WT precipitated enrichment of LINC01783 and DLL-1 (Fig. 4b). Moreover, luciferase reporter assays displayed that overexpression of miR-432-5p hindered luciferase activity of LINC01783-WT but failed to have effects on LINC01783-Mut (Fig. 4c). The similar results could be seen in the luciferase activity of DLL-1-3′UTR-WT as well as that of DLL-1-3′UTR-Mut, the overexpression of miR-432-5p significantly impaired the luciferase activity of DLL-1-3′UTR-WT rather than that of DLL-1-3′UTR-Mut (Fig. 4d). Therefore, miR-432-5p bound to LINC01783 or DLL-1 through the predicted binding sites. Afterwards, we noticed that miR-432-5p overexpression inhibited luciferase activity of DLL-1-3′UTR-WT, but then up-regulation of LINC01783 offset the impacts of miR-432-5p up-regulation on DLL-1-3′UTR-WT luciferase activity (Fig. 4e). To summarize, LINC01783 modulates miR-432-5p to modulate DLL-1 expression.

### LINC01783 accelerates the proliferation and migration of NSCLC cells by targeting miR-432-5p/DLL-1

**Fig. 5:**
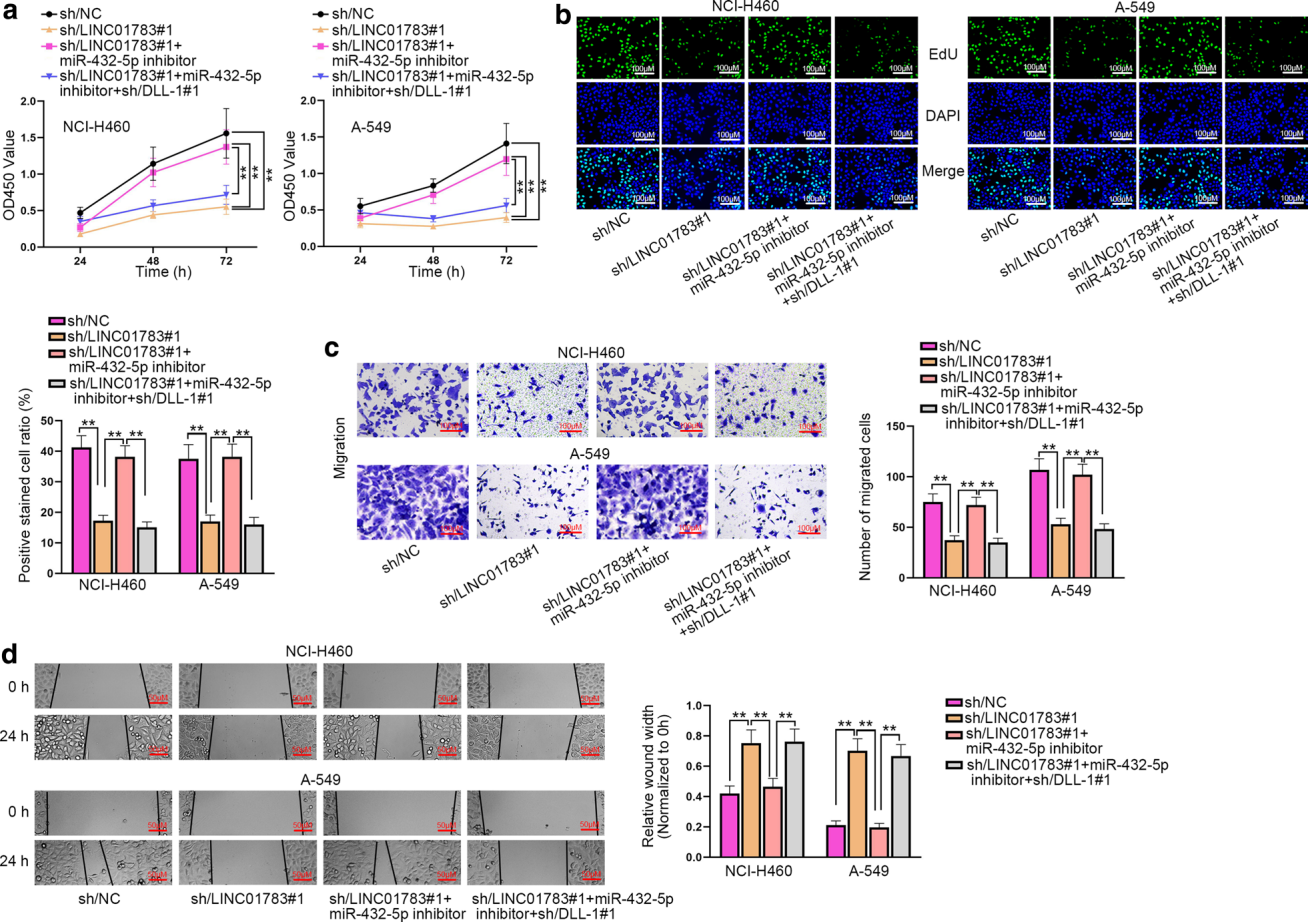
LINC01783 accelerates the proliferation and migration of NSCLC cells by targeting miR-432-5p/DLL-1. **a**–**d** CCK-8 (**a**), EdU (**b**), transwell (**c**) and wound healing (**d**) assays were conducted for detecting cell proliferation (**a**, **b**) and migration (**c**, **d**) in NCI-H460 and A-549 cells with sh/NC, sh/LINC01783#1, miR-432-5p inhibitor and sh/DLL-1#1 (One-way ANOVA, Tukey). **P < 0.01

Finally, we performed rescue assays to confirm the regulatory mechanism of the LINC01783/miR-432-5p/DLL-1 axis. Firstly, knockdown efficiency of DLL-1 was verified via Western blot assay (Additional file 1: Figure S1G). Next, we performed Western blot assay again and found that silenced miR-432-5p could rescue down-regulated DLL-1 caused by LINC01783 ablation, but then DLL-1 depletion offset the rescue (Additional file 1: Figure S1H). To further explore the mechanism, we subsequently conducted the functional experiments. Data exhibited that miR-432-5p depletion could reverse the inhibitory effect of silenced LINC01783 on cell proliferation, but then DLL-1 silence countervailed the effects imposed by miR-432-5p down-regulation in CCK-8 and EdU assays (Fig. 5a, b). Meanwhile, the falling migration and invasion induced by down-regulated LINC01783 was restored by miR-432-5p depletion and then DLL-1 silencing recovered the suppressive effects on cell migration and invasion induced by LINC01783 knockdown in transwell and wound healing assays (Fig. 5c, d, Additional file 1: Figure S1I). Taken together, LINC01783 boosts the progression of NSCLC cells by targeting miR-432-5p/DLL-1.

## Discussion

Emerging studies have suggested that lncRNAs exerted crucial functions in the development of various cancers. For instance, DLX6-AS1 accelerated the process of cervical cancer through sponging miR-16-5p to modulate ARPP19 [14]. NEAT1 triggered cell proliferation and migration in colon cancer by inhibiting miR-185-5p and elevating IGF2 [15]. FAM83H-AS1 contributed to the development of triple-negative breast cancer via sponging miR-136-5p to enhance metadherin [16]. A large number of studies have suggested the closely connection between lncRNAs and malignancies. NSCLC, a type of common cancer with high morbidity and mortality has been verified to be affected by various lncRNAs. For instance, SNHG1 promoted NSCLC progression through sponging miR-497 [17]. Likely, lncRNA PTAR accelerates NSCLC development as a sponge of miR-101, promoting cell proliferation, migration and invasion [10]. Moreover, SNHG16 facilitated the process of NSCLC via modulating EphA2 and miR-520a-3p [18]. Therefore, the study on relationship between lncRNAs and NSCLC was reasonable.

LINC01783 has been identified as an oncogene in the process of cervical cancer through targeting miR-199b-5p/GBP1 [13]. However, the impact of LINC01783 on other cancers has not been clearly manifested. The previous studies have demonstrated that miR-432-5p function in glioma [19], lung adenocarcinoma [20]. Given the fact that miR-432-5p has been proved to be related with NSCLC, sponged by lncRNA MSTRG.51053.2 or Linc00668 to affect the progression of NSCLC [21, 22], we deciphered and studied the potential ceRNA relationship between LINC01783 and miR-432-5p.

In addition, former study has indicated that DLL-1 elevates antitumor T-cell immunity and could be used as a target for lung cancer treatment [23].

CeRNA network was the main mechanism filed to be investigated when it comes to the function of lncRNAs in diseases [24]. Because the regulation of post-transcription requires lncRNA to locate mainly in the cytoplasmic fraction of cancer cells [25], LINC01783 was determined to have ceRNA feature by FISH assays after the test of its function in NSCLC cells. In our study, LINC01783 was determined to regulate DLL-1 through targeting miR-432-5p in NSCLC cells. We identified that LINC01783 functioned in NSCLC cells via Notch pathway, positively related to the expression of DLL-1. Furthermore, down-regulated DLL-1 restored the effects of LINC01783 knockdown on the proliferation, migration and invasion of NSCLC cells. To sum up, LINC01783 targets miR-432-5p to modulate DLL-1 expression and activate Notch pathway in NSCLC cells. Additionally, the promoting function has been verified through in vivo assays. Our present study is the first exploration on the effect of LINC01783 on NSCLC cell progression and identifies a novel potential target LINC01783 for the treatment of NSCLC. For the treatment, the transcription of LINC01783 could be inhibited by the loss of transcription factor. Meanwhile, Notch pathway could also be regulated by interfering the translation or transcription of its essential proteins or genes. Hence, the upstream and downstream mechanisms of LINC01783 in NSCLC cells need a penetrating research. However, the influence of the mechanism in this study on clinicopathological features of NSCLC patients has not been proved. The verification of clinicopathological relevance will be fulfilled in our further studies on the molecular mechanism of LINC01783/miR-432-5p/DLL-1 axis in NSCLC patient clinical samples.

## Supplementary Information

**Additional file 1: Figure S1.** (A) QPCR was conducted to analyze the effect of Ad12SV40 on the expression level of LINC01783 in BEAS-2B cells (Student’s t-test). (B) Transwell assays were conducted to examine the invasive capacity of NCI-H460 and A-549 cells after transfection with sh/LINC01783#1/2 (One-way ANOVA, Dunnett). (C) In vivo experiments were used to explore the effect of LINC01783 on NSCLC tumor growth (Student’s t-test). (D) Western Blot assay was used to analyze the Notch pathway-related proteins of the NSCLC cells after transfection with sh/LINC01783#1/2. (E) Transwell assays were conducted to test whether DLL-1 could reverse the loss of invasive capacity induced by silencing of LINC01783 (One-way ANOVA, Tukey). (F) The mutated or normal binding sites between LINC01783, DLL-1 and miR-432-5p were shown. (G) Western Blot assay was used to verify the knockdown efficiency of sh/DLL-1#1/2/3. (H) Western Blot assay detected the protein level of DLL-1 in the transfected NCI-H460 and A-549 cells in the rescue experiments. (I) Transwell assays were conducted to examine the invasive capacity of NCI-H460 and A-549 cells after the indicated transfections in the rescue experiments (One-way ANOVA, Tukey). **P < 0.01.

## Data Availability

Not applicable.

## Abbreviations

NSCLC: Non-small cell lung canner
lncRNAs: Long non-coding RNAs
ceRNA: Competing endogenous RNA
mRNAs: Messenger RNAs
miRNAs: MicroRNAs
LINC01783: Long intergenic non-protein coding RNA 1783
DLL-1: Delta-like 1
ATCC: American Type Culture Collection
FBS: Fatal Bovine Serum
RT-qPCR: Real-time quantitative polymerase chain reaction
CCK-8: Cell counting kit-8
EdU: 5-Ethynyl-2′-deoxyuridine
DAPI: 4′,6-Diamidino-2-phenylindole
FISH: Fluorescence in situ hybridization
RIP: RNA immunoprecipitation
WT: Wild-type
Mut: Mutant
SD: Standard deviation
ANOVA: Analysis of variance
