# Supplementary figures and images for: LINC01783 facilitates cell proliferation, migration and invasion in non-small cell lung cancer by targeting miR-432-5p to activate the notch pathway
Source: Cancer Cell Int. 2021 Apr 26;21:234. doi: 10.1186/s12935-021-01912-0 (PMC8073972; doi:10.1186/s12935-021-01912-0)

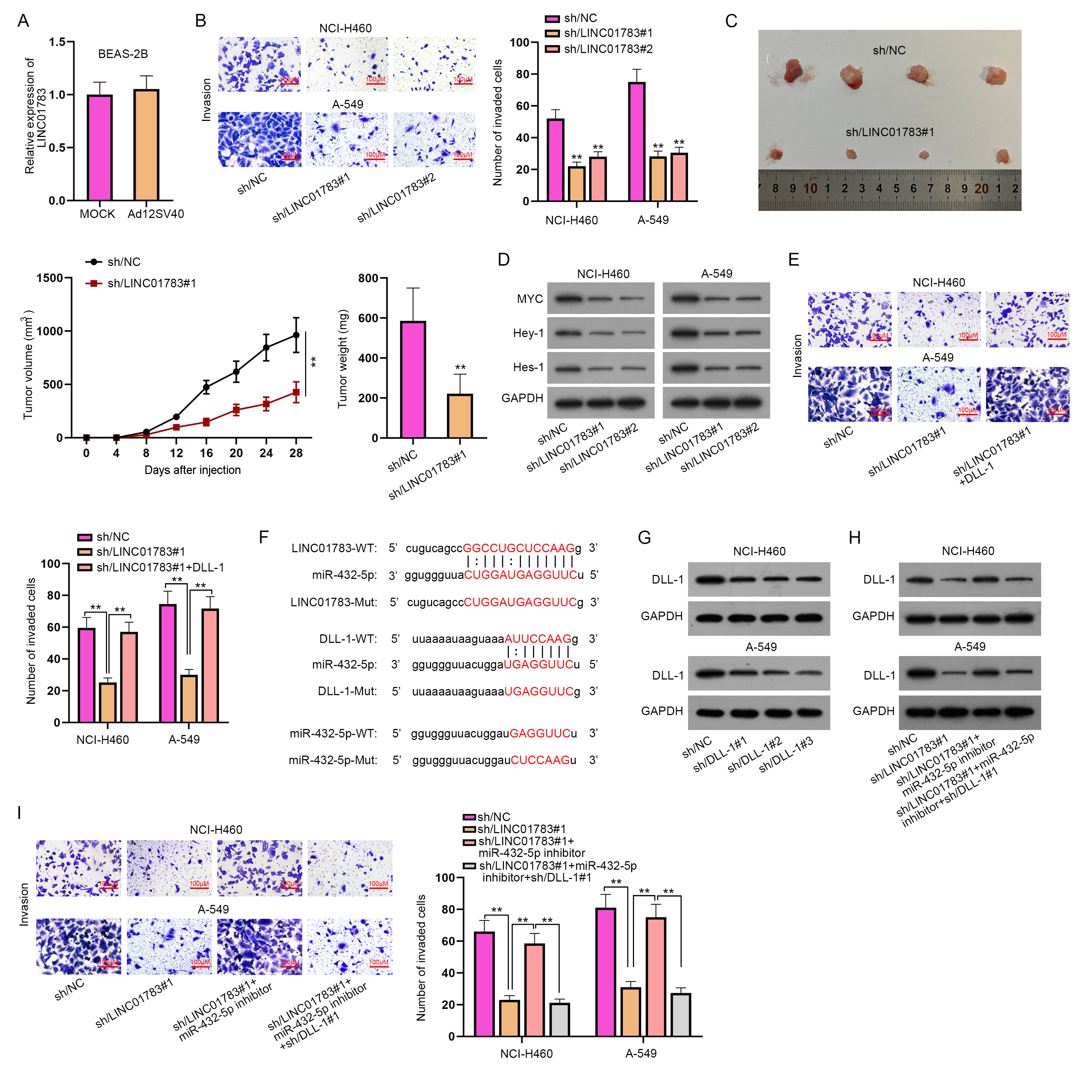

Supplement: Supplementary file 1 — Additional file 1: Figure S1. (A) QPCR was conducted to analyze the effect of Ad12SV40 on the expression level of LINC01783 in BEAS-2B cells (Student’s t-test). (B) Transwell assays were conducted to examine the invasive capacity of NCI-H460 and A-549 cells after transfection with sh/LINC01783#1/2 (One-way ANOVA, Dunnett). (C) In vivo experiments were used to explore the effect of LINC01783 on NSCLC tumor growth (Student’s t-test). (D) Western Blot assay was used to analyze the Notch pathway-related proteins of the NSCLC cells after transfection with sh/LINC01783#1/2. (E) Transwell assays were conducted to test whether DLL-1 could reverse the loss of invasive capacity induced by silencing of LINC01783 (One-way ANOVA, Tukey). (F) The mutated or normal binding sites between LINC01783, DLL-1 and miR-432-5p were shown. (G) Western Blot assay was used to verify the knockdown efficiency of sh/DLL-1#1/2/3. (H) Western Blot assay detected the protein level of DLL-1 in the transfected NCI-H460 and A-549 cells in the rescue experiments. (I) Transwell assays were conducted to examine the invasive capacity of NCI-H460 and A-549 cells after the indicated transfections in the rescue experiments (One-way ANOVA, Tukey). **P < 0.01. [file 12935_2021_1912_MOESM1_ESM.tif]
